# A phase I study to evaluate the effect of high-dose carbidopa on levodopa pharmacokinetics

**DOI:** 10.3389/fphar.2025.1596139

**Published:** 2025-07-07

**Authors:** Akane Hayashi, Atsushi Hosomi, Shinsaku Kihara, Masato Nishi, Saori Torisawa, Yoshiumi Ouchi, Hidenori Takada, Junichi Enokizono

**Affiliations:** ^1^ Research Management Office, Research Planning Department, Research Division, Kyowa Kirin Co., Ltd., Tokyo, Japan; ^2^ Pharmacokinetics Research Laboratories, Research Unit, Research Division, Kyowa Kirin Co., Ltd., Shizuoka, Japan; ^3^ Clinical Science Department, Development Division, Kyowa Kirin Co., Ltd., Tokyo, Japan; ^4^ Clinical Development Center, Development Division, Kyowa Kirin Co., Ltd., Tokyo, Japan; ^5^ Biometrics Department, Development Division, Kyowa Kirin Co., Ltd., Tokyo, Japan; ^6^ Medical Pharmacology Department, Development Division, Kyowa Kirin Co., Ltd., Tokyo, Japan

**Keywords:** Parkinson’s disease, phase I clinical trial, high-dose carbidopa combination, levodopa half-life prolongation, continuous dopamine stimulation

## Abstract

Levodopa is the gold standard treatment for Parkinson’s disease. However, a high unmet medical need exists for longer-lasting oral levodopa formulations to achieve sustained motor improvement with reduced risk of the effect wearing off. In our previous non-clinical studies using rats, more than 3-fold prolongation of the levodopa half-life was achieved when it was combined with a high carbidopa dose. This study aimed to evaluate the effects of high-dose carbidopa on the pharmacokinetics of levodopa. A phase I study was performed to examine the effects of a combination of levodopa, carbidopa, and entacapone in healthy male volunteers. Levodopa and entacapone doses were set at approved dose levels. The carbidopa dose ranged from the approved 10 mg–600 mg. In addition to plasma concentrations of levodopa, those of the metabolites were also determined to evaluate their inhibitory effects on levodopa-metabolizing enzymes. The plasma concentrations of levodopa and its metabolites were monitored by liquid chromatography-tandem mass spectrometry up to 24 h after administration. The observed adverse effects were mild, and all participants completed the study. At higher carbidopa doses, the area under the levodopa plasma concentration-time curve increased by approximately 2-fold, and the half-life of levodopa was slightly prolonged by < 1.4-fold. These changes were much smaller than those observed in rats. The ratios of levodopa metabolites suggested that dopa deoxycarboxylase inhibition was saturated at a carbidopa dose of 300 mg. In conclusion, higher carbidopa doses are tolerable, and the effect of carbidopa on levodopa half-life is limited.

**Clinical trial registration:**

https://jrct.mhlw.go.jp/en-latest-detail/jRCT2051200104, registry number jRCT2051200104.

## 1 Introduction

Levodopa (LD), the most effective drug for Parkinson’s disease treatment, has poor oral availability; therefore, it is taken concomitantly with inhibitors of LD-metabolizing enzymes, including dopa deoxycarboxylase (DDC) and catechol-O-methyltransferase (COMT) (Gershanik, 2015). Carbidopa (CD) and benserazide are clinically available DDC inhibitors, whereas entacapone (ET) and opicapone are clinically available COMT inhibitors. Although LD oral availability is much improved by co-administration with DDC and COMT inhibitors, its half-life (*t*_1/2_) remains short (approximately 1.5 h), and LD is usually taken three times daily (Gershanik, 2015). The short *t*_1/2_ of LD causes large fluctuations in the plasma concentration, which might be a risk factor for the unfavorable adverse effects of LD, including effect wearing off and dyskinesia (Chase et al., 1989; Nutt et al., 2000; Olanow et al., 2000). Multiple approaches, including sustained release formulations, have been used to prolong elevated plasma LD concentrations, sustain stable efficacy, and avoid adverse effects. Sustained release formulations of LD and CD (Sinemet^®^ CR and Rytary^®^) and of LD, CD, and ET (Stalevo^®^) provide longer drug duration by slowing the intestinal absorption rate; however, the elimination *t*_1/2_ of LD is not altered (Hsu et al., 2015). The effectiveness of sustained-release formulations is limited by the short transit time of the drugs in the gastrointestinal tract (2–6 h).

Long-term continuous administration of a combined LD and CD (DUOPA^®^) formulation is possible through a tube placed in the intestine via the stomach using a portable pump (Olanow et al., 2014). This drug formulation achieves a sustained increased plasma LD concentration for approximately 16 h. However, placing the tube requires invasive surgery. Therefore, this drug is indicated only for patients with advanced Parkinson’s disease.

Considering these limitations, there is an unmet medical need for long-lasting LD formulations that do not require invasive surgery. Prolonging the elimination *t*_1/2_ of LD is an ideal strategy, because it provides an enhanced plasma LD concentration via oral administration for an extended duration without limiting the gastrointestinal transit time. In our previous non-clinical studies, LD elimination *t*_1/2_ was prolonged by co-administration of LD with a very high dose of CD. As a result, in rats, LD elimination *t*_1/2_ increased from 0.766 h to 3.45 h (a 4.5-fold increase) when the CD dose was changed from 2.5 mg/kg to 300 mg/kg. The maximum plasma concentration (C_max_) of CD (given at a dose of 2.5 mg) was approximately 40 ng/mL, which was comparable to C_max_ of CD observed in humans at an approved CD dose (10 mg). The C_max_ of 300 mg/kg CD was approximately 2,000 ng/mL in rats. We hypothesized that LD elimination *t*_1/2_ would be considerably prolonged by administering CD at doses higher than the currently approved dose. Therefore, we designed an open-label phase I study of a new LD, CD, and ET formulation to verify this hypothesis. The LD and ET doses were set at the approved dose levels of 100 mg or 200 mg, respectively. The CD dose was increased from 10 mg to 600 mg. In addition, plasma concentrations of LD metabolites were measured to evaluate the inhibitory effect of the new combined formulation on DDC and COMT.

## 2 Materials and methods

### 2.1 Ethical conduct

The study protocol and informed consent forms were reviewed and approved by the Institutional Review Board of the investigation site, the Medical Corporation Heishinkai (no: 1088PB). Written informed consent was obtained from all participants. This clinical study complied with relevant laws and regulations, including the Pharmaceutical and Medical Device Act.

### 2.2 Study design

**FIGURE 1:**
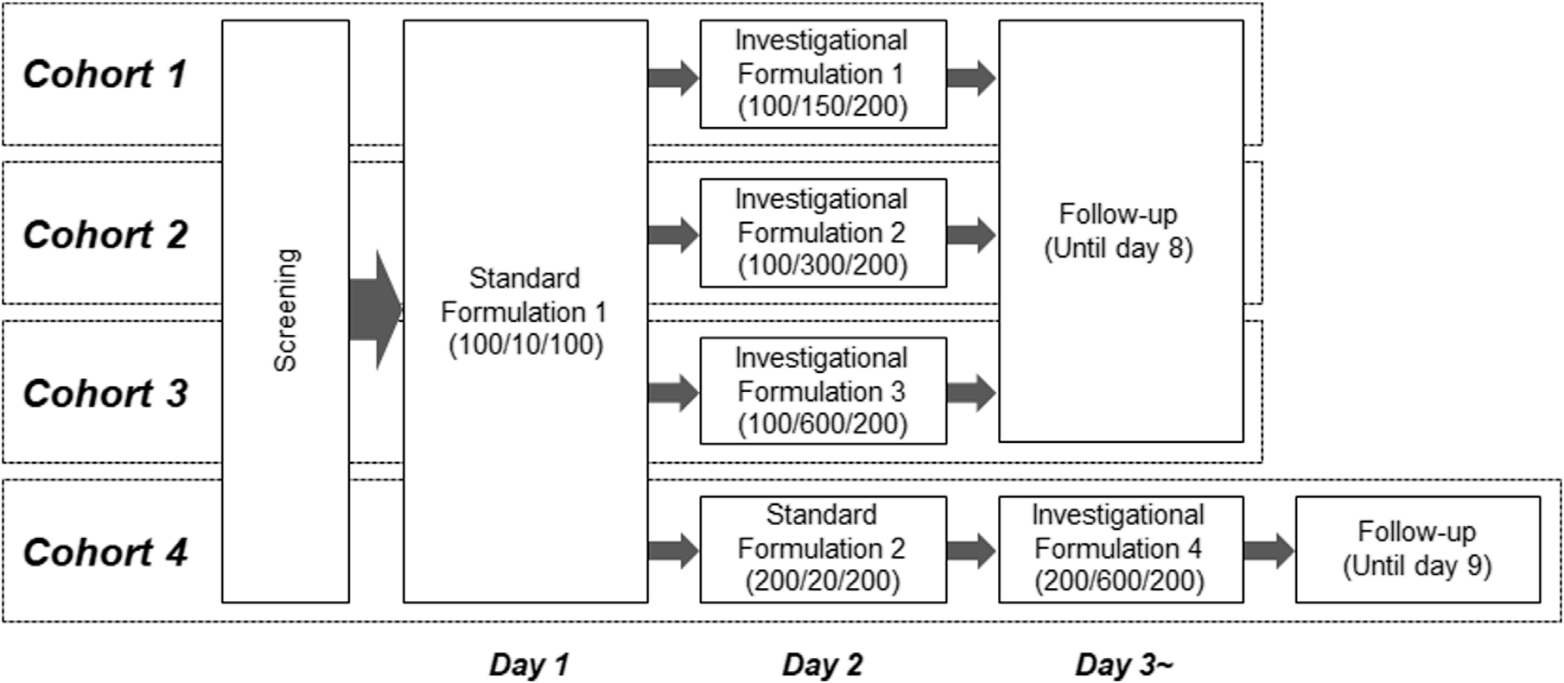
Schematic representation of the clinical study design. Values in parentheses for standard or investigational formulations indicate combination doses (mg) of levodopa/carbidopa/entacapone.

This was an open-label, single-dose, phase I study of a combination of LD, CD, and ET, with increasing doses of CD in healthy male volunteers. The primary objective of this study was to evaluate the effect of an increased CD dose on the pharmacokinetic profile of LD. The study design is shown in Figure 1. Participants underwent screening tests within 28 days after informed consent was obtained, and those confirmed to be eligible were enrolled in four cohorts (n = 8/cohort). In each cohort, the participants received a standard formulation, which contained an approved combination of LD/CD/ET doses, and an investigational formulation, in which the CD dose exceeded the approved dose after a 24-h interval. A sufficient washout period was ensured owing to the short *t*_1/2_ of LD, CD, and ET (only several hours).

The combined LD/CD/ET doses of the standard formulations were determined with reference to a commercial drug (Stalevo^®^) that contains 100/10/100 mg of LD/CD/ET in each tablet, with one or two tablets taken at a time. Therefore, the LD/CD/ET combination doses were set at 100/10/100 mg (standard formulation 1) and 200/20/200 mg (standard formulation 2). For cohorts 1–3, the LD and ET doses were set to 100 mg and 200 mg, respectively, and the CD dose was increased to 150 mg (investigational formulation 1), 300 mg (investigational formulation 2), or 600 mg (investigational formulation 3). In cohort 4, the combination doses of LD/CD/ET for investigational formulation 4 were set at 200/600/200 mg to evaluate the effect of the maximum CD dose on the pharmacokinetics of an increased LD dose. In cohort 4, participants received standard formulation 1, standard formulation 2, and investigational formulation 4 at 24-h intervals. Blood was sampled before administration and at 0.25, 0.5, 0.75, 1, 1.5, 2, 3, 4, 5, 6, 8, 10, and 12 h after administration of the standard formulations. For the investigational formulations, blood samples were collected at the same time points as for the standard formulations, with an additional sample at 24 h. Plasma was prepared using 1,2-diaminoethane-N, N, N′, N′-tetraacetic acid dipotassium salt as anti-coagulant.

During the follow-up phase, the participants underwent a check-up and were discharged after the absence of abnormalities was confirmed. Follow-up periods were 8 days for cohorts 1‒3 and 9 days for cohort 4.

### 2.3 Test formulations

Two commercial drugs (DOPACOL^®^ L100, 100 mg LD and 10 mg CD per tablet; and AMEL^®^, 100 mg ET per tablet) were used for the standard formulations. For the investigational formulations, granules of CD hydrate were prepared and used as an additional CD source. The granules contained 42% w/w CD and excipients, including lactose hydrate, low-substituted hydroxypropyl cellulose, hydroxypropyl cellulose, and magnesium stearate.

### 2.4 Quantitative measurements of LD, CD, and ET

Plasma concentrations of LD, CD, and ET were measured using liquid chromatography-tandem mass spectrometry (LC-MS/MS). The LD and CD concentrations were measured simultaneously under the same analytical conditions, whereas the ET concentration was measured separately. A Triple Quad 6,500 system (AB Sciex, MA, United States) was used for the LC-MS/MS analysis.

For LD and CD analysis, plasma samples (10 μL) and ultra-pure water (10 μL) were mixed with ice-cold internal standard (IS)-containing solution [75 ng/mL L-Dopa-(phenyl-d3) and 10 ng/mL (S)-(−)-CD-d3 in a solution of 3 mmol/L ammonium formate, 0.1% formic acid, and 90% acetonitrile]. The mixed solutions were centrifuged (3,100 × g, 4°C, 10 min), and 80 μL of the supernatant was evaporated to dryness under a nitrogen gas stream. The dried samples were reconstituted in 80 μL of the mobile phase (1 vol% acetonitrile containing 0.2 vol% formic acid) and then centrifuged (3,100 × g, 4°C, 10 min), and the supernatants were subjected to LC-MS/MS analysis. To prepare calibration standards, plasma samples containing LC and CD were processed in the same manner. LD, CD, and their ISs were separated on a reverse-phase column (AQUITY UPLC BEH Phenyl, 1.7 μm, 2.1 mm I.D. × 150 mm, Waters, MA, United States) in isocratic mode with the mobile phase. The flow rate was 0.35 mL/min, and the column temperature was 20°C. The analytes and ISs were subjected to positive electrospray ionization and monitored in the multiple-reaction monitoring mode. The mass-to-charge (m/z) ratios of the monitored ions were 198.1/107.0 (Q1/Q3) for LD, 227.0/181.1 for CD, 201.0/110.0 for L-Dopa-(phenyl-d3), and 230.0/184.0 for (S)-(−)-CD-d3.

For ET analysis, plasma samples (10 μL) were mixed with 190 μL of ice-cold IS containing methanol (10 ng/mL ET-d10) and then centrifuged (3,100 × g, 4°C, 10 min). The supernatants (100 μL) were mixed with 0.1% formic acid (100 μL) and subjected to LC-MS/MS analysis. To prepare calibration standards, plasma containing ET was processed in a similar manner. ET and ISs were separated on a reverse-phase column (ZORBAX SB-C18, 5 μm, 2.1 mm I.D. × 50 mm, Agilent Technologies, CA, United States) in the gradient mode with 0.1% formic acid (A) and 0.1% formic acid in acetonitrile (B). The flow rate was 0.45 mL/min, and the column temperature was 40 °C. The concentration of mobile phase B (%) was altered linearly in the following order: 35% (0 min), 35% (0.3 min), 85% (1.4 min), 98% (1.41 min), 98% (2.50 min), 35% (2.51 min), and 35% (4.00 min). The analyte and IS were subjected to positive electrospray ionization and monitored in the multiple-reaction monitoring mode. The m/z ratios of the monitored ions were 306.0/233.0 (Q1/Q3) for ET, and 316.0/233.0 for ET-d10.

The peak area ratios of the analyte and corresponding IS (y-axis) were plotted against the analyte concentrations (x-axis). Calibration curves were obtained using linear least-squares regression with a weighting factor of 1/x^2^. The concentration ranges used for quantification were 20–10,000 ng/mL for LD, 4–2,000 ng/mL for CD, and 5–2,000 ng/mL for ET. To measure plasma samples with analyte concentrations above the upper limit of quantification, plasma samples were diluted with blank plasma and processed as described above.

### 2.5 Quantitative measurement of 3,4-dihydroxyphenylacetic acid (DOPAC), dopamine sulfate (DA-S), and 3-O-methyldopa (3-OMD)

The plasma concentrations of DOPAC, DA-S, and 3-OMD were determined using LC-MS/MS. DA-S and 3-OMD were measured simultaneously, while DOPAC was measured separately. For DA-S and 3-OMD, plasma samples (20 μL) were mixed with 75 μL of ice-cold solution (0.1% formic acid/acetonitrile/trichloroacetic acid [85/10/5, v/v/v]) containing deuterium-labeled ISs for the two analytes (50 ng/mL each). After centrifugation (5,000 × g, 4°C, 10 min), the supernatants (50 μL) were mixed with 0.1% formic acid (75 μL) and subjected to LC-MS/MS analysis. The analytes and ISs were separated on a reverse-phase column (Unison UK-C18, 3 μm, 50 mm I.D. × 2 mm, Imtakt, Kyoto, Japan) using an LC system (Nexera X_2_ system, Shimazu, Kyoto, Japan) in a gradient mode with 0.2% formic acid (A) and acetonitrile (B). The flow rate was 0.35 mL/min, and the column temperature was 40 °C. The percentage of mobile phase B was linearly altered as follows: 1% (0–0.5 min), 70% (1.5 min), 90% (1.51–2 min), and 1% (2.01–3.5 min). The MS/MS analysis was performed using a Triple Quad 6,500 system. The analytes and ISs were subjected to negative electrospray ionization and monitored in multiple-reaction monitoring mode. The m/z ratios of the monitored ions were 234.1/137.2, 212.1/166.0 for 3-OMD, 238.1/141.2 for DA-S-d_4_, and 215.1/156.1 3-OMD-d_3_.

For DOPAC, plasma samples (20 μL) were mixed with ice-cold methanol (75 μL) containing DOPAC-d5 (50 ng/mL of). After centrifugation (5,000 × g, 4°C, 10 min), the supernatants (50 μL) were mixed with 0.1 vol% formic acid (75 μL) and subjected to the LC-MS/MS analysis. The analyte and IS were separated on a reverse-phase column (XBridge Phenyl 3.5 μm, 2.1 mm I.D. × 50 mm, Waters) using an LC system (Nexera X_2_ system) in a gradient mode with 0.2% formic acid (A) and methanol containing 0.1% formic acid (B). The flow rate was 0.35 mL/min, and the column temperature was 35°C. The percentage of mobile phase B was linearly altered as follows: 5% (0–0.1 min), 10% (1 min), 55% (2 min), 85% (4 min), and 5% (4.01–5 min). The MS/MS analysis was performed using an API4000 system (AB Sciex). The analyte and IS were subjected to negative electrospray ionization and monitored in multiple-reaction monitoring mode. The m/z ratios of the monitored ions were 166.9/123.1 for DOPAC and 172.1/128.1 DOPAC-d_5_.

### 2.6 Pharmacokinetic analysis

The C_max_ and time to reach C_max_ were determined from the plasma concentration-time profile. The elimination rate constant (*k*_el_) was determined from the slope of the log-linear elimination phase of the plasma concentration-time profile. The *t*_1/2_ value was determined as ln2/*k*_el_. The area under the plasma concentration-time curve from time zero to the last measurable time point (AUC_0–*t*_) was determined using the linear trapezoidal method. The area under the plasma concentration-time curve from time zero to infinity ( ${\text{AUC}}_{0–\infty }$ ) was determined using the following equation, where C_*t*_ is the plasma concentration at the last measurable time point.

$${\text{AUC}}_{0-\infty }={\text{AUC}}_{0-t}+\frac{C_{t}}{k_{\text{el}}}$$

The apparent clearance (CL/F) was determined by dividing the dose by the ${\text{AUC}}_{0–\infty }$ . The apparent distribution volume was determined by dividing CL/F by *k*_el_.

The metabolic ratios of the three LD metabolites were determined by dividing the AUC of each metabolite by that of LD.

## 3 Results

### 3.1 Enrolled participants and tolerability

All 32 enrolled participants (n = 8/cohort) have completed the study. The mean age (±standard deviation) of participants was 25.8 ± 4.56 years, the mean body weight was 64.93 ± 5.324 kg, and the mean body mass index was 21.38 ± 1.440 kg/m^2^. All the parameters were similar among the four cohorts.

Treatment-emergent adverse effects (TEAEs) were observed in 7/32 participants (21.9%) and included decreased appetite, headache, and decreased frustration tolerance in cohort 1; insomnia and nosebleed in cohort 2; hypertriglyceridemia and skin rash in cohort 3; and abdominal distension in cohort 4. All the TEAEs were mild, and only one instance of each TEAE was observed. No abnormalities in the vital signs or electrocardiogram results were observed. Therefore, a single dose of all investigational formulations was confirmed to be tolerable.

### 3.2 Effect of increased CD on LD pharmacokinetics

**FIGURE 2:**
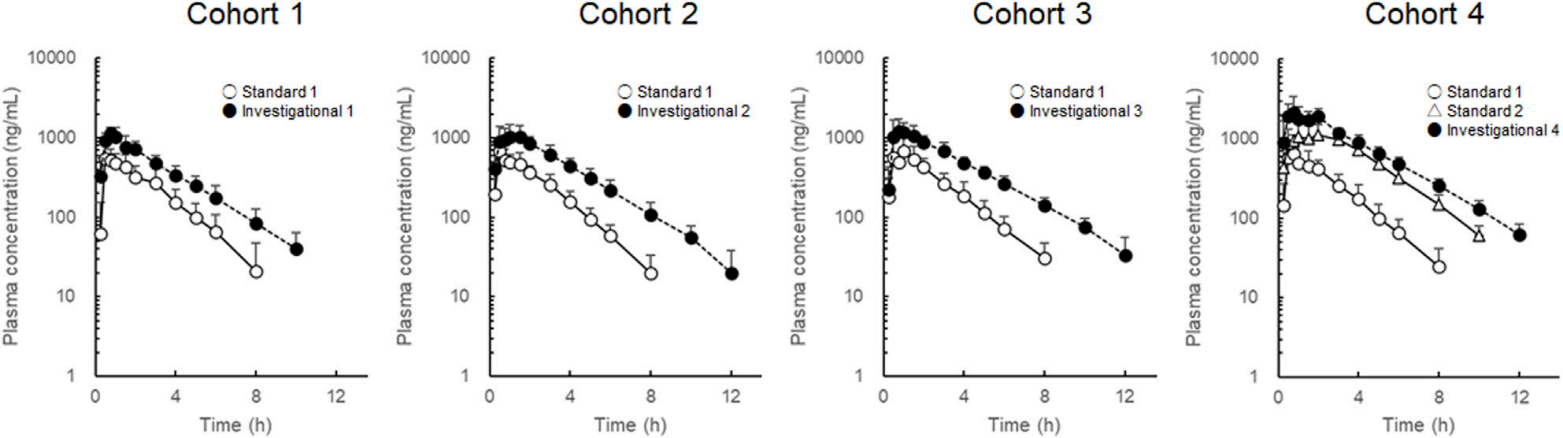
Plasma concentration-time profiles of levodopa after administration of the combined levodopa, carbidopa, and entacapone formulations. Each symbol with a bar represents the mean + standard deviation, n = 8.

To avoid inter-individual variation, each participant received standard and investigational formulations, and changes in LD pharmacokinetics were evaluated for each cohort. LD plasma concentration-time profiles are shown in Figure 2, and LD pharmacokinetic parameters are summarized in Table 1. In cohorts 1–3, where the LD dose was fixed at 100 mg, LD ${\text{AUC}}_{0–\infty }$ values after administration of the investigational formulations were increased compared with those after administration of standard formulation 1. The fold-changes were comparable, although they showed a slight increase with increasing CD dose. LD *t*_1/2_ values after administration of the investigational formulations showed a prolonged trend compared with those after administration of standard formulation 1. The fold *t*_1/2_ changes were 1.18 ± 0.14, 1.34 ± 0.13, and 1.35 ± 0.13 in cohorts 1, 2, and 3, respectively. Compared with cohort 1, fold changes of *t*_1/2_ in cohorts 2 and 3 were slightly higher.

**TABLE 1:** Pharmacokinetic parameters of levodopa.

Cohort	Formulation or ratio	*t*_max_ (h)	C_max_ (ng/mL)	AUC_0–*t*_ (ng·h/mL)	${\text{AUC}}_{0–\infty }$ (ng·h/mL)	*t*_1/2_ (h)	CL/F (L/h)	MRT (h)
Cohort 1	Standard formulation 1	0.88 (0.50–1.50)	709 ± 179	1,560 ± 496	1,650 ± 503	1.58 ± 0.16	65.4 ± 17.1	2.69 ± 0.50
	Investigational formulation 1	0.75 (0.50–1.00)	1,240 ± 206	3,470 ± 807	3,550 ± 830	1.84 ± 0.14	29.3 ± 5.6	3.06 ± 0.39
	Ratio	0.875 (0.500–1.50)	1.83 ± 0.45	2.28 ± 0.30	2.21 ± 0.27	1.18 ± 0.14	0.458 ± 0.056	1.16 ± 0.17
Cohort 2	Standard formulation 1	0.63 (0.50–1.50)	787 ± 187	1,650 ± 256	1,710 ± 259	1.48 ± 0.12	59.7 ± 9.1	2.54 ± 0.38
	Investigational formulation 2	0.75 (0.50–3.00)	1,370 ± 352	4,130 ± 680	4,230 ± 675	1.97 ± 0.13	24.2 ± 4.0	3.35 ± 0.50
	Ratio	1 (0.500–3.00)	1.87 ± 0.89	2.52 ± 0.35	2.49 ± 0.32	1.34 ± 0.13	0.407 ± 0.048	1.34 ± 0.22
Cohort 3	Standard formulation 1	1 (0.50–2.00)	848 ± 323	1,860 ± 460	1,930 ± 469	1.61 ± 0.14	55.0 ± 16.1	2.66 ± 0.42
	Investigational formulation 3	1 (0.50–3.00)	1,560 ± 343	4,720 ± 709	4,860 ± 728	2.17 ± 0.19	21.1 ± 3.7	3.68 ± 0.35
	Ratio	1 (0.333–3.00)	2.05 ± 0.84	2.61 ± 0.39	2.58 ± 0.37	1.35 ± 0.13	0.394 ± 0.055	1.41 ± 0.25
Cohort 4	Standard formulation 1	0.75 (0.50–1.50)	781 ± 284	1,720 ± 467	1,790 ± 465	1.51 ± 0.16	60.9 ± 22.7	2.63 ± 0.34
	Standard formulation 2	1.75 (0.50–3.00)	1,500 ± 312	5,370 ± 934	5,460 ± 944	1.64 ± 0.13	37.9 ± 8.6	3.46 ± 0.27
	Investigational formulation 4	1.13 (0.50–2.00)	2,620 ± 1,047	8,460 ± 1,576	8,660 ± 1,622	2.04 ± 0.25	23.9 ± 4.9	3.53 ± 0.33
	Ratio	0.875 (0.500–1.50)	1.76 ± 0.60	1.58 ± 0.12	1.59 ± 0.11	1.25 ± 0.14	0.632 ± 0.046	1.02 ± 0.08

Abbreviations: AUC_0–*t*_, area under the plasma concentration-time curve from time zero to the last measurable time point; ${\text{AUC}}_{0–\infty }$ , area under the plasma concentration-time curve from time zero to infinity; CL/F, apparent clearance; C_max_, maximum plasma concentration; MRT, mean residence time; *t*_max_, time to reach the maximum concentration; *t*_1/2_, half-life.

In cohort 4, LD C_max_ and ${\text{AUC}}_{0–\infty }$ values after administration of standard formulation 1 were comparable with those in cohorts 1–3. The LD dose of investigational formulation 4 was 200 mg. After administration of investigational formulation 4, LD ${\text{AUC}}_{0–\infty }$ and *t*_1/2_ values were higher than those after administration of standard formulation 2. The fold changes were 1.59 ± 0.12 for the ${\text{AUC}}_{0–\infty }$ and 1.25 ± 0.14 for the *t*_1/2_.

The pharmacokinetic parameters of CD and ET are listed in Table 2. The C_max_ and ${\text{AUC}}_{0–\infty }$ values of CD increased almost dose-dependently within the dose range of 10–300 mg. Although C_max_ and ${\text{AUC}}_{0–\infty }$ values after 600 mg CD were larger than those for 300 mg CD, the increases were sublinear. CD *t*_1/2_ values tended to be longer at higher doses. For ET, the C_max_ and AUC_0–*t*_ values were dose-dependent. The *t*_1/2_ values could not be determined for all participants; therefore, the ${\text{AUC}}_{0–\infty }$ , CL/F, and mean residence time values were not determined.

**TABLE 2:** Pharmacokinetic parameters of carbidopa and entacapone.

Carbidopa								
Cohort	Formulation	*t*_max_ (h)	C_max_ (ng/mL)	AUC_0–*t*_ (ng•h/mL)	AUC_0–∞_ (ng•h/mL)	*t*_1/2_ (h)	CL/F (L/h)	MRT (h)
Cohort 1	Standard formulation 1	3 (1.50-4.00)	47.5 ± 24.3	206 ± 125	221 ± 125	1.71 ± 0.18	60.3 ± 35.5	4.35 ± 0.42
	Investigational formulation 1	3 (2.00-3.00)	618 ± 285	3,090 ± 1,611	3,150 ± 1,622	2.15 ± 0.65	57.5 ± 24.1	4.38 ± 0.40
Cohort 2	Standard formulation 1	3 (1.50-4.00)	40.3 ± 15.5	193 ± 78	208 ± 80	1.60 ± 0.17	55.6 ± 23.7	4.21 ± 0.30
	Investigational formulation 2	3 (3.00-4.00)	1470 ± 420	7,600 ± 2,134	7,640 ± 2,124	3.25 ± 0.65	43.2 ± 16.5	4.97 ± 0.39
Cohort 3	Standard formulation 1	3.5 (2.00-5.00)	37.4 ± 12.7	176 ± 70	190 ± 70	1.68 ± 0.14	62.0 ± 31.2	4.37 ± 0.51
	Investigational formulation 3	3 (3.00-4.00)	1,760 ± 479	10,200 ± 2,848	10,200 ± 2,889	3.75 ± 0.31	63.2 ± 19.3	5.07 ± 0.34
Cohort 4	Standard formulation 1	2.5 (2.00-4.00)	48.7 ± 14.4	200 ± 56	216 ± 57	1.72 ± 0.19	49.2 ± 13.2	4.35 ± 0.45
	Standard formulation 2	3 (3.00-4.00)	89.6 ± 33.5	438 ± 158	456 ± 160	1.87 ± 0.22	48.6 ± 16.5	4.86 ± 0.32
	Investigational formulation 4	3 (3.00-4.00)	1,790 ± 440	10,100 ± 2,949	10,200 ± 2,944	3.91 ± 0.65	64.2 ± 21.6	5.21 ± 0.43

Entacapone				
Cohort	Formulation	*t*_max_ (h)	C_max_ (ng/mL)	AUC_0–*t*_ (ng•h/mL)
Cohort 1	Standard formulation 1	0.75 (0.50–3.00)	574 ± 272	827 ± 167
	Investigational formulation 1	0.75 (0.50–3.00)	832 ± 263	1,480 ± 255
Cohort 2	Standard formulation 1	0.88 (0.50–3.00)	521 ± 246	855 ± 176
	Investigational formulation 2	0.75 (0.25–4.00)	1,120 ± 321	1860 ± 368
Cohort 3	Standard formulation 1	0.88 (0.50–3.00)	575 ± 191	839 ± 168
	Investigational formulation 3	1.5 (0.75–3.00)	858 ± 336	1800 ± 406
Cohort 4	Standard formulation 1	0.5 (0.50–3.00)	581 ± 277	843 ± 223
	Standard formulation 2	0.88 (0.50–4.00)	834 ± 220	1800 ± 430
	Investigational formulation 4	0.5 (0.50–5.00)	981 ± 606	1720 ± 596

Abbreviations: AUC_0–*t*_, area under the plasma concentration-time curve from time zero to the last measurable time point; ${\text{AUC}}_{0–\infty }$ , area under the plasma concentration-time curve from time zero to infinity; CL/F, apparent clearance; C_max_, maximum plasma concentration; MRT, mean residence time; *t*_max_, time to reach the maximum concentration; *t*_1/2_, half-life.

### 3.3 Pharmacokinetics of the three metabolites

The metabolic pathway of LD is shown in the Supplementary Figure. LD is metabolized to dopamine by DDC and 3-OMD by COMT. Dopamine is further metabolized to multiple metabolites including DA-S by sulfotransferase and DOPAC by monoamine oxidase. To evaluate the effect of increased CD dose on the enzymatic activities of DDC and COMT, concentrations of DOPAC, DA-S, and 3-OMD were measured in plasma samples obtained from cohorts 1–3. The ${\text{AUC}}_{0–\infty }$ values and metabolic ratios of the three metabolites are shown in Table 3. The metabolic ratios of DOPAC and DA-S after administration of the investigational formulations were lower than those after administration of standard formulation 1 in cohorts 1–3. For DOPAC, the percentage reduction in the metabolic ratio increased with increasing CD doses. The percentage of reduction was 90.7% at the highest CD dose. For DA-S, the percentage reductions in the metabolic ratios were 70.9% in cohort 1 and approximately 77% in cohorts 2 and 3. However, the metabolic ratios of 3-OMD were comparable between standard formulation 1 and investigational formulations. These results suggested that the inhibitory effects of CD on DDC were dose-dependent, whereas the inhibitory effects on COMT remained unchanged when ET was increased from 100 to 200 mg.

**TABLE 3:** Metabolic ratios of the three levodopa metabolites.

		DOPAC			DA-S			3-OMD		
Cohort	Formulations	${\text{AUC}}_{0–\infty }$ (ng•h/mL)	Metabolic ratio	Reduction (%)	${\text{AUC}}_{0–\infty }$ (ng•h/mL)	Metabolic ratio	Reduction (%)	${\text{AUC}}_{0–\infty }$ (ng•h/mL)	Metabolic ratio	Reduction (%)
Cohort 1	Standard formulation 1	364 ± 76	0.285	-	1,330 ± 170	0.739	-	5,880 ± 2,280	3.31	-
	Investigational formulation 1	239 ± 52	0.0837	70.6	891 ± 166	0.215	70.9	11,700 ± 3,500	3.07	7.25
Cohort 2	Standard formulation 1	490 ± 98	0.337	-	1,450 ± 310	0.730	-	5,860 ± 1,290	3.22	-
	Investigational formulation 2	215 ± 60	0.0626	81.4	801 ± 109	0.167	77.1	14,600 ± 3,500	3.24	−0.62
Cohort 3	Standard formulation 1	429 ± 96	0.278	-	1,330 ± 340	0.606	-	6,340 ± 1,970	3.08	-
	Investigational formulation 3	101 ± 46	0.0259	90.7	780 ± 156	0.137	77.4	17,100 ± 3,400	3.30	−7.14

Abbreviations: ${\text{AUC}}_{0–\infty }$ , area under the plasma concentration-time curve from time zero to infinity; DA-S, dopamine sulfate; DOPAC, 3,4-dihydroxyphenylacetic acid; 3-OMD, 3-O-methyldopa.

## 4 Discussion

After oral administration, LD dissolves in gastrointestinal tract, is absorbed there, and after undergoing first-pass metabolism in liver, reaches systemic blood. LD in systemic blood is cleared by metabolism mainly in liver and kidney. All of these processes affect the pharmacokinetics of LD; however, the dissolution rate and systemic clearance mainly contribute to the duration of plasma LD concentration. Sustained release formulations of LD (Sinemet^®^ CR and Rytary^®^, and Stalevo^®^) achieved prolonged duration of plasma LD concentration (Hsu et al., 2015). However, the effect is limited by the transit time of LD and the combination drugs in gastrointestinal tract. For further prolongation of plasma LD concentration, delaying the systemic clearance of LD is needed. Our previous non-clinical studies suggested that the therapeutic plasma concentrations of carbidopa (200–400 nmol/L) are not sufficient to delay the systemic clearance of LD because the liver concentrations of DDC is much higher (submicromolar to micromolar range). Supraclinical dose of carbidopa is needed to prolong the *t*_*1/2*_ of LD.

In this study, we evaluated the effect of a CD dose exceeding the approved level on LD pharmacokinetics. The increased CD dose was expected to ameliorate the fluctuation of the plasma LD concentration, which could prolong pharmacological activity and reduce unfavorable events, such as wearing-off of the drug effect and dyskinesia. The CD dose was increased to 600 mg, which is 30–60 times higher than the approved doses. One concern was the safety risk. However, all observed TEAEs were mild. Among TEAEs, decreased frustration tolerance and skin rash were identified as drug-related effects. However, no trend in CD dose-dependency was observed for these adverse effects. Increased CD doses were tolerable up to at least a single dose. These results suggest that substantially higher CD doses were tolerable.

Comparisons of LD plasma concentrations following administrations of the standard and investigational formulations showed that in cohorts 1–3, LD ${\text{AUC}}_{0–\infty }$ values after administration of the investigational formulations were more than 2-fold higher than those after administration of standard formulation 1. Compared with standard formulation 1, in addition to an increase in the CD dose, the ET dose was increased from 100 mg to 200 mg. Therefore, the effects of an increased ET dose had to be considered. To evaluate the contribution of higher CD and ET doses to increased plasma exposure to LD, the inhibitory effects on DDC and COMT were evaluated based on the metabolic ratios of three LD metabolites. The metabolic ratios of 3-OMD were not different in cohorts 1–3, suggesting that standard formulation 1 and the investigational formulations inhibited COMT similarly. However, the metabolic ratios of DA-S and DOPAC were lower following administration of investigational formulations compared with those after treatment with standard formulation 1, suggesting stronger inhibition of DDC by the investigational formulations. These results suggested that the increased LD ${\text{AUC}}_{0–\infty }$ was mainly caused by the stronger inhibition of DDC by the higher CD dose. The fold changes in LD ${\text{AUC}}_{0–\infty }$ and *t*_1/2_ values for the investigational formulations were similar between cohorts 2 and 3. The percentage reductions in the metabolic ratios of DOPAC and DA-S were also similar between cohorts 2 and 3. These results suggest that the inhibitory effects of CD on DDC were mostly saturated at a CD dose of 300 mg.

A longer *t*_1/2,_ which would reduce fluctuations in LD plasma concentration, is thought to be important for producing sustainable pharmacological activity and reducing unfavorable adverse effects. In our previous studies using rats, LD *t*_1/2_ was prolonged by increasing the CD dose, whereas increasing the ET dose did not prolong LD *t*_1/2_. Following administration of CD at a dose of 300 mg/kg, LD *t*_1/2_ was prolonged by approximately 3-fold and CD C_max_ was >1,000 ng/mL, which was more than 10-fold higher than CD C_max_ after administration of the highest approved CD dose. Those results motivated us to conduct the present clinical study. For the CD dose of 600 mg in investigational formulations 3 and 4, CD C_max_ reached approximately 1,800 ng/mL. Although LD ${\text{AUC}}_{0–\infty }$ increased more than 2-fold with investigational formulation 3 in cohort 3, the increase in LD *t*_1/2_ was only 1.35-fold. Similar results were obtained in cohort 4. An interspecies difference was observed between humans and rats in the effect of an increased CD dose on LD *t*_1/2_. Based on the reduced metabolic ratios of DOPAC and DA-S, the inhibitory effects of CD on DDC were saturated at a CD dose of 600 mg. Similar results were observed in rats treated with 300 mg/kg CD. Another potential reason for interspecies differences could be the varying contribution of COMT to LD clearance. If the contribution of COMT were higher in humans than in rats, the effect of an increased CD dose on LD *t*_1/2_ would be limited, even if DDC was completely inhibited. Opicapone, a new COMT inhibitor, is currently used in clinical practice. Opicapone inhibited COMT, increased plasma exposure to LD, and suppressed 3-OMD production stronger that ET did (Rocha et al., 2013; Rocha et al., 2014). A combination of a higher CD dose and opicapone could potentially prolong LD *t*_1/2_.

This was a phase 1 study, and the participant number was limited in each cohort. Considering the detection sensitivity with the limited participant number, we designed the study to evaluate the effect of high-dose carbidopa under homogeneous condition as possible. Therefore, only healthy male volunteer was recruited. However, gender difference should be considered to evaluate the effect on the pharmacokinetics of LD. After oral administration of LD with DDC or COMT inhibitors, plasma exposure of LD is higher in women than in men (Kompoliti et al., 2002; Kumagai et al., 2014; Nishikawa et al., 2020; Conti et al., 2022). Several possible reasons explains the gender difference. In women, gastric emptying time is slower (Datz et al., 1987), and COMT activity in liver is approximately 25% lower than in men (Boudíková et al., 1990). Aging is also reported to affect the plasma exposure of LD (Contin et al., 1991; Nishikawa et al., 2020). To conclude the effect of high-dose carbidopa, study with broader population is needed. We planned such broader population study in phase 2; however, we decided not to proceed to phase 2 because the result of the present study was negative.

In conclusion, different from the result of non-clinical studies, high-dose carbidopa showed only small effect on LD *t*_*1/2*_ in human at least in healthy male volunteer. Higher contribution of COMT in human is a possible reason. If it is true, combination with higher dose entacapone or more potent COMT inhibitor would improve LD *t*_*1/2*_. Recently, an intraoral micropump device for continuous LD delivery was developed (Murch et al., 2024). The device allows continuous LD delivery from oral cavity in a non-invasive way, and did not affect gastrointestinal transit time. In the clinical study involving patients with Parkinson disease, LD and CD administration with the device achieved nearly flat plasma concentration of LD and significantly reduced OFF time compared to conventional oral tablets of LD and CD (Olanow et al., 2024). The absorption phase still offers room to improve the duration of plasma LD concentration using such promising delivery technology.

## Data Availability

The original contributions presented in the study are included in the article/Supplementary Material, further inquiries can be directed to the corresponding author.
