# Supplementary material for: A phase I study to evaluate the effect of high-dose carbidopa on levodopa pharmacokinetics
Source: Front Pharmacol. 2025 Jul 7;16:1596139. doi: 10.3389/fphar.2025.1596139 (PMC12277756; doi:10.3389/fphar.2025.1596139)
Supplement: Supplementary file 1 [file Image1.pdf]

Supplementary Material

Supplementary Figures

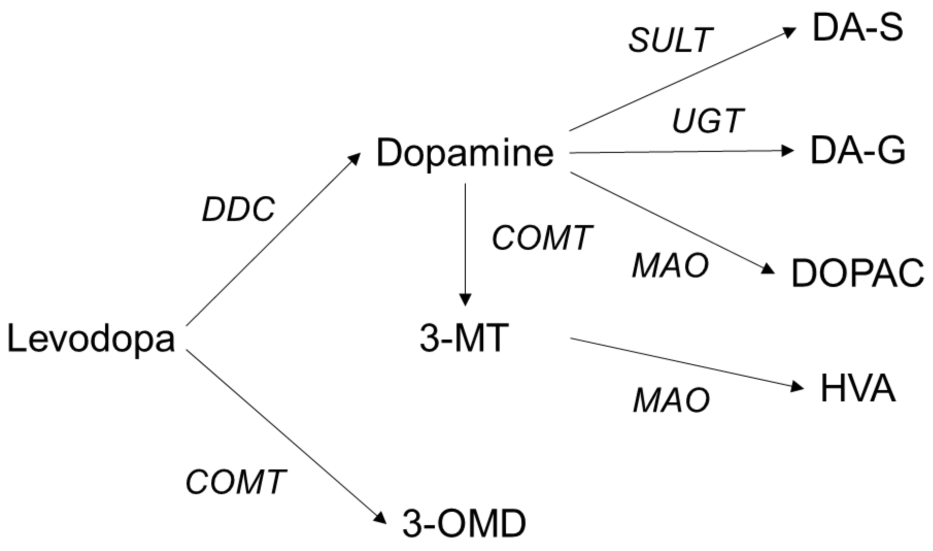

Supplementary Figure 1. Metabolic pathway of Levodopa. DDC, dopa deoxycarboxylase; COMT, catechol-O-methyltransferase; MAO, monoamine oxidase; SULT, sulfotransferase; UGT, UDP-glucuronosyltransferase; DA-S, dopamine sulfate; DA-G, dopamine-4β-glucuronide; DOPAC, 3,4-dihydroxyphenylacetic acid; 3-OMD, 3-O-methyldopa; 3-MT, 3-methoxytyramine; HVA, homovanillic acid.
